# NPBWR1 and NPBWR2: Implications in Energy Homeostasis, Pain, and Emotion

**DOI:** 10.3389/fendo.2013.00023

**Published:** 2013-03-18

**Authors:** Takeshi Sakurai

**Affiliations:** ^1^Kanazawa UniversityKanazawa, Japan

**Keywords:** neuropeptide B, neuropeptide W, hypothalamus, limbic system, amygdala, pain, emotions

## Abstract

Neuropeptide B/W receptor-1 (NPBWR1) and NPBWR2 had been known as orphan receptors GPR7 and 8, respectively. Endogenous peptide ligands of these receptors, neuropeptide B (NPB) and neuropeptide W (NPW), were identified in 2002 and 2003 (Fujii et al., 2002; Brezillon et al., 2003; Tanaka et al., 2003). These peptides have been implicated in regulation of feeding behavior, energy homeostasis, neuroendocrine function, and modulating inflammatory pain. In addition, strong and discrete expression of their receptors in the extended amygdala and bed nucleus of the stria terminalis suggests a potential role in regulating stress responses, emotion, anxiety, and fear. Recent studies of NPB/NPW using both pharmacological and phenotypic analyses of genetically engineered mice as well as a human study support this hypothesis.

## Introduction

Receptors for neuropeptide B (NPB)/neuropeptide W (NPW) were originally identified as orphan receptors GPR7 and GPR8, which share 70% nucleotide identity and 64% amino acid identity with each other. They have relatively high similarities with opioid and somatostatin receptors. GPR8 was not found in the rodent genomes, while GPR7 was highly conserved in both human and rodents (O’Dowd et al., 1995). This suggests the GPR8 gene was relatively recently generated through gene duplication.

In 2002–2003, two endogenous peptide ligands for these receptors were identified and named NPB/NPW (Fujii et al., 2002; Shimomura et al., 2002; Brezillon et al., 2003; Tanaka et al., 2003). Following the deorphaning of these receptors, GPR7 and GPR8 were reclassified by IUPHAR as NPB/W receptor-1 (NPBWR1) and NPB/W receptor-2 (NPBWR2), respectively (Davenport and Singh, 2005).

In rodents, *Npbwr1* mRNA is localized in some discrete brain regions, including the hypothalamus (dorsomedial hypothalamus and suprachiasmatic nucleus), hippocampus, ventral tegmental area (VTA), and extended amygdala (CeA and bed nucleus of the stria terminalis; BNST) (Lee et al., 1999; Tanaka et al., 2003), suggesting this receptor is involved in regulatory mechanisms of homeostasis, reward system, and emotion.

This review discusses recent findings concerning the pharmacology, histology, and phenotypic analysis of genetically engineered mice of the NPB/NPW system.

## Identification of NPB and NPW

In 2002–2003, three groups independently identified endogenous peptide ligands for GPR7 and GPR8 by so-called “reverse pharmacology” in combination with bioinformatics approaches. To identify the cognate endogenous ligands for GPR7 (NPBWR1) and GPR8 (NPBWR2), Shimomura et al. (2002) expressed these receptors in Chinese Hamster Ovary (CHO) cells and measured the decrease in forskolin-induced cAMP production in these cells as the read-out for receptor activation. While screening the bovine hypothalamic extract fractions, they detected activities that specifically inhibit cAMP production in those cells, and purified the activities. During their purification process, they identified two forms of NPW with different peptide lengths of 23 and 30 amino acid residues, and named them NPW23 and NPW30, respectively.

In a similar manner, three groups (Fujii et al., 2002; Brezillon et al., 2003; Tanaka et al., 2003) independently purified and identified an additional endogenous ligand for NPBW1 and NPBW2. Fujii et al. first screened the Celera database to identify novel secretory peptides and then expressed the cDNAs of the putative secretory peptides to find novel peptide ligands. Subsequent pharmacological studies and purification of the peptide from bovine hypothalamic extracts led to identification of the second ligand for GPR7 and GPR8, which was named NPB due to the unique modification, namely, bromination of the first tryptophan residue. Brezillon et al. (2003) also identified human NPB mRNA using a bioinformatics approach by searching the EST database using the NPW sequence as a query.

Tanaka et al. used a unique melanin-pigment aggregation assay in *Xenopus* melanophore cells expressing GPR7 as the assay system to purify NPB from bovine hypothalamus. Through EST database searches they also identified NPW as a putative paralogous peptide (Tanaka et al., 2003).

## Structures of NPW and NPB

**Figure 1:**
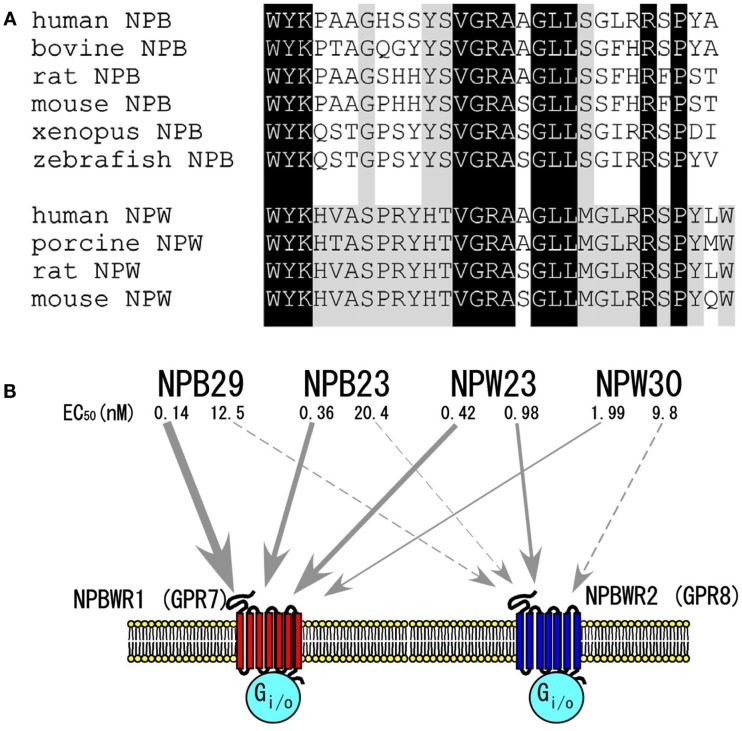
**The NPB/NPW system**. **(A)** Amino acid sequences of NPB and NPW. Dark shadow shows amino acid identity between NPB and NPW. Light shadow shows conserved amino acids within NPB or NPW. **(B)** NPB/W and their receptors. EC_50_ values were determined by assay of inhibition of cAMP production in CHO cells expressing each receptor (Brezillon et al., 2003). Modified from Hondo et al. (2008).

Neuropeptide W and NPB do not display any significant sequence similarity to members of other known peptide families, while sharing a high degree of sequence similarity with each other, constituting a distinct family of peptides (Figure 1A) (Fujii et al., 2002; Shimomura et al., 2002; Brezillon et al., 2003; Tanaka et al., 2003).

As mentioned earlier, sequence analysis of purified peptides showed that NPW has two isoforms with lengths of 23 and 30 amino acid residues, i.e., neuropeptide W23 (NPW23) and neuropeptide W30 (NPW30), respectively. NPW23 is produced as a result of proteolytic processing at a pair of arginine residues in the 24th and 25th positions in NPW30 (Fujii et al., 2002; Brezillon et al., 2003; Tanaka et al., 2003). *In vitro* experiments using cells expressing these receptors showed that synthetic NPW23 activates and binds to both NPBWR1 and NPBWR2 at similar effective doses, while NPW30 shows slightly lower affinity to both receptors as compared with NPW23 (Figure 1B) (Brezillon et al., 2003; Tanaka et al., 2003).

As mentioned earlier, NPB has a unique modification at the N-terminus tryptophan residue, C-6-bromination (Fujii et al., 2002; Tanaka et al., 2003). While this represents the first evidence of bromination of the protein in mammals, the biological significance of this modification is unclear, as it has been demonstrated that des-bromo-NPB is equipotent to brominated NPB in *in vitro* cAMP inhibition assays (Tanaka et al., 2003). By analogy with NPW, Brezillon et al. (2003) predicted that two isoforms of NPB, NPB23 and NPB29, could be produced from the processing of a 125-amino acid human precursor through the alternative usage of a dibasic amino acid pair. However, the dibasic motif, Arg24–Arg25, which is seen in human NPB, does not exist in NPB of other mammalian species including bovine, rat, and mouse. Therefore, it is unlikely that the NPB23 isoform exists as a mature peptide in non-human species. Consistently, both Fujii et al. and Tanaka et al. were only able to isolate NPB29 from bovine hypothalamus extracts during their purification procedures.

In *X. laevis* melanophore pigment aggregation assay, NPB29 binds and activates human NPBWR1 or NPBWR2 with median effective concentrations (EC_50_) of 0.23 and 15.8 nM, respectively (Tanaka et al., 2003), suggesting that NPB is a relatively selective agonist for NPBWR1 (Figure 1B).

The preferred conformations of des-bromo-NPB and -NPW have been determined by a combination of ^1^H NMR, CD, and molecular modeling (Lucyk and Miskolzie, 2005).

## Structure-Activity Relationships of NPW and NPB

The rank order of potency of NPB and NPW isoforms has been determined in cell lines expressing NPBWR1 and NPBWR2. Both NPW and NPB bind and activate NPBWR1 and NPBWR2 receptors with varying degrees of affinity. NPBWR1 has slightly higher affinity for NPB as compared with both forms of NPW, whereas NPBWR2 shows a potency rank order of NPW23 > NPW30 > NPB (Figure 1B) (Fujii et al., 2002; Shimomura et al., 2002; Brezillon et al., 2003; Tanaka et al., 2003). NPB23 and NPB29 have similar affinity for both receptors. NPW23 and NPW30 bind to both receptors almost equally, while NPB acts as a relatively selective agonist for NPBWR1.

Tanaka et al. (2003) showed that deletion of Trp-1 from NPB or NPW markedly decreased their activity, suggesting that the N-terminus is involved in receptor binding. This is consistent with the fact that NPB and NPW have similarity in their sequences in the N-terminal region.

## Structures and Functions of NPBWR1 and NPBWR2

**Figure 2:**
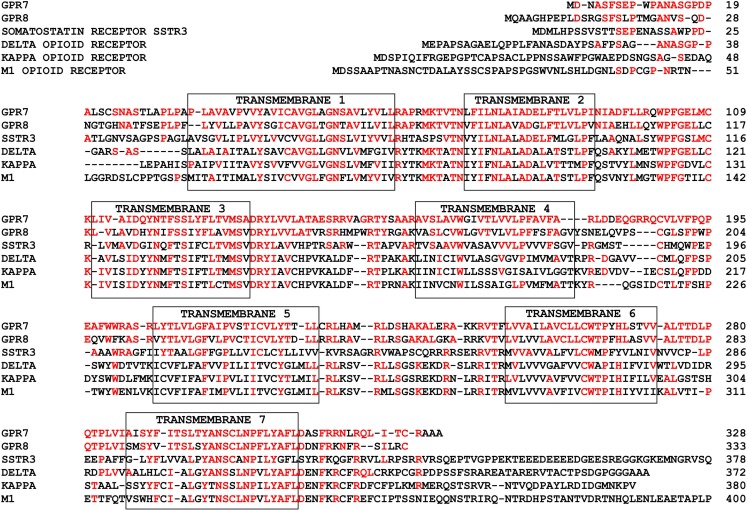
**Primary structures of NPBWR1/NPBWR2 compared to somatostatin and opioid receptors**. Conserved amino acid residues are shown in red. Each transmembrane domain is boxed. Modified from Hondo et al. (2008).

The human NPBWR1 and NPBWR2 genes are localized on chromosome 10q11.2–121.1 and 20q13.3, respectively. Human NPBWR1 and NPBWR2 have 328 and 333 amino acid residues, respectively, and share 64% sequence homology with each other (Figure 2). Among other family members of GPCRs, NPBW1 and NPBW2 are most closely related to opioid and somatostatin receptors (Figure 2) (O’Dowd et al., 1995). Amino acid analysis of NPBW1 orthologs in other mammalian species has revealed a high degree of conservation throughout evolution (Lee et al., 1999). In contrast, while the gene encoding NPBWR2 has been discovered in several mammalian species such as monkey, lemur, bat, shrew, and rabbit, it has not been found in rodent genomes (Lee et al., 1999), suggesting that these two receptors were produced by relatively phylogenetically recent gene duplication (Lee et al., 1999).

As suggested by the assay systems used during their purification process and structural similarities to somatostatin/opioid receptors, both NPBWR1 and NPBWR2 couple to the Gi-class of G-proteins (Tanaka et al., 2003). This suggests that these neuropeptides have inhibitory properties on neurons via activation of GIRK (Kir3) channels. NPB and NPW were also shown to stimulate Erk p42/p44 activity in human adrenocortical carcinoma-derived NCI-H295 cells (Andreis et al., 2005). This activation is probably mediated by beta/gamma subunits released from Gi-proteins (Tim van et al., 1995). Recently, a synthetic low molecular weight antagonist for NPBWR1 has been reported (Anthony Romero et al., 2012).

## Distribution of NPB/NPW and NPBWR1/NPBWR2 in Brain and Other Tissue

### Neuropeptide B

**Figure 3:**
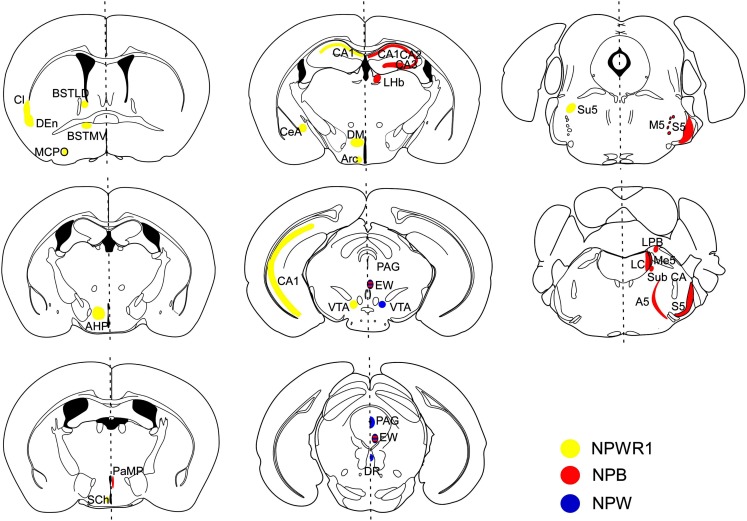
**Schematic representation of distribution of NPB/NPW and *Npbwr1* mRNA in mouse brain coronal sections**. Receptor distribution is shown in the right hemisphere, while ligand distribution is shown in the left. *Npb* mRNA (red regions) was observed in the hippocampus (CA1, CA2, CA3), lateral habenular nucleus (LHb), paraventricular hypothalamic nucleus, medial parvicellular part (PaMP), Edinger–Westphal (EW) nucleus, motor root of the trigeminal nerve (m5), sensory root of the trigeminal nerve (s5), lateral parabrachial nucleus alpha part (Sub CA), locus coeruleus (LC), noradrenergic cell group A5 (A5), and inferior olive subnucleus B (IOB) (Tanaka et al., 2003). *Npw* mRNA (blue regions) was observed in the periaqueductal gray (PAG) matter, EW nucleus (EW), ventral tegmental area (VTA), and dorsal raphe nucleus (DR). NPBWR1 mRNA (yellow regions) was observed in the claustrum (Cl), dorsal endopiriform nucleus (DEn), bed nucleus of the stria terminals, laterodorsal part (BSTLD), bed nucleus of the stria terminalis, medioventral part (BSTMV), suprachiasmatic nucleus (Sch), magnocellular preoptic nucleus (MCPO), paraventricular hypothalamic nucleus, posterior part (PaPo), dorsomedial hypothalamic nucleus (DM), central amygdala (CeA), CA1 field, hippocampus (CA1), ventral tegmental area (VTA), sensory root trigeminal nerve (Su5), subiculum (S), anterior hypothalamic area, posterior part (AHP), and arcuate hypothalamic nucleus (Arc). Modified from Hondo et al. (2008).

*Npb* mRNA was found in several specific regions in the mouse brain such as the paraventricular hypothalamic nucleus (PVN), CA1–CA3 fields of the hippocampus, and several nuclei in the midbrain and brainstem, including the Edinger–Westphal (EW) nucleus, the sensory and motor nuclei of the trigeminal nerve, locus coeruleus (LC), inferior olive, and lateral parabrachial nucleus (Figure 3) (Tanaka et al., 2003; Jackson et al., 2006).

Schulz et al. reported that NPB-immunoreactive cell bodies were observed in many regions within the hypothalamus. High levels of *Npbwr1* and *Npb* mRNA expression were also observed in the hypothalamus, including the ventromedial hypothalamic nucleus, dorsomedial hypothalamic nucleus, arcuate nucleus, supraoptic retrochiasmatic nucleus, and in the area ventral to the zona incerta (Schulz et al., 2007). Although *Npb* mRNA was detected in several regions outside the hypothalamus such as the hippocampus and brain stem (Tanaka et al., 2003; Jackson et al., 2006; Schulz et al., 2007), the existence of NPB-immunoreactive neurons in these regions in the brain has not been clearly demonstrated, since no good antibody for NPB is available.

In peripheral tissues, expression of human *Npb* mRNA was detected by RT-PCR in the kidney, uterus, ovary, testis, and placenta, while murine *Npb* mRNA was detected by Northern blot at high levels in the stomach, spinal cord, and testis and at lower levels in the liver and kidney (Brezillon et al., 2003). The biological significance of this expression remains unknown.

### Neuropeptide W

The existence of NPW in the mouse brain was more confined to several nuclei in the midbrain and brainstem including the EW, VTA, periaqueductal gray (PAG), and dorsal raphe nucleus (DR) (Figure 3) (Tanaka et al., 2003; Kitamura et al., 2006). Most of these neurons expressed tyrosine hydroxylase (TH), suggesting that these cells are also catecholaminergic (our unpublished observation). In humans, high levels of *Npw* mRNA were detected in the substantia nigra, and moderate expression levels were detected in the amygdala and hippocampus (Fujii et al., 2002). In peripheral tissues, expression of human *Npw* mRNA was confirmed by RT-PCR in the progenital system, comprising the kidney, testis, uterus, ovary, and placenta, and also in the stomach and respiratory system, while murine *Npw* mRNA was detected by Northern blot at high levels in the lung and lower levels in the stomach (Brezillon et al., 2003).

Consistent with the mRNA distribution, NPW-immunoreactive (ir) cells were also exclusively detected in the EW, VTA, PAG, and DR in rats (Kitamura et al., 2006). NPW-ir fibers were observed in several brain regions in rats including the lateral septum, bed nucleus of the stria terminalis (BNST), dorsomedial and posterior hypothalamus, CeA, CA1 field of hippocampus, interpeduncular nucleus, inferior colliculus, lateral parabrachial nucleus, facial nucleus, and hypoglossal nucleus. Among these regions, NPW-ir fibers were most abundantly observed in the CeA and BNST, the output nuclei of the extended amygdala. These observations suggest that NPW-producing neurons are exclusively localized to the mid brain, and they project mainly to the limbic system, especially the CeA and BNST (Figure 3).

Some reports showed the existence of NPW-ir cell bodies in the PVN in rats and mice (Dun et al., 2003). However, the staining of NPW-like immunoreactivity-positive cells in the PVN is likely to be non-specific staining, for two main reasons (Kitamura et al., 2006). First, PVN immunoreactivity is also observed in *NPW*^−/−^ mice using many of the commercially available antibodies. Second, *Npw* mRNA is not expressed in the PVN in either mice or rats (Kitamura et al., 2006).

### NPBWR1 (GPR7)

*In situ* hybridization study showed that the CeA and BNST express the highest levels of NPBWR1 mRNA expression in the mouse brain (Figure 3) (Tanaka et al., 2003; Singh et al., 2004; Jackson et al., 2006). Other nuclei with high levels of *Npbwr1* are the suprachiasmatic (SCN) and ventral tuberomammillary nuclei of the hypothalamus. Moderate levels of expression are seen in the CA1–CA3 regions of the hippocampus, dorsal endopiriform, dorsal tenia tecta, bed nucleus, and red nucleus. Low level expression of *Npbwr1* is seen in the olfactory bulb, parastrial nucleus, hypothalamus, laterodorsal tegmentum, superior colliculus, LC, and nucleus of the solitary tract.

### NPBWR2 (GPR8)

Because *Npbwr* 2 does not exist in rodent genomes, very limited information about the tissue distribution of NPBWR2 has been available thus far. RT-PCR analysis showed that *Npbwr2* mRNA is strongly expressed in the human amygdala and hippocampus. Lower levels of expression were also detected in the corpus callosum, cerebellum, substantia nigra, and caudate nucleus (Brezillon et al., 2003).

## Biological Activities of NPB and NPW

Several papers reported pharmacological actions of NPB and NPW *in vivo* (Table 1). In this section, I discuss biological activities of these peptides, referring the pharmacological actions and phenotypes of genetically-modified mice on NPB/W systems (Table 2).

**Table 1:** ***In vivo* pharmacological effects of NPB/NPW**.

Substance	Effects	Animal	Preference
NPW (i.c.v.)	Food intake ↑	Rat (male)	Shimomura et al. (2002)
	Body weight ↑		Tanaka et al. (2003)
NPW (i.c.v.)	Body temperature ↑	Rat	Mondal et al. (2003)
	Heat production ↑	
NPW	ACTH ↑	Rat	Hochol et al. (2007)
	Estradiol ↑	
NPW30 (i.c.v.)	Arterial blood pressure (ABP) ↑	Rat	Yu et al. (2007)
	Heart rate (HR) ↑	
	Plasma catecholamine concentration ↑	
NPB (i.c.v.)	Food intake (light period–dark period) ↑	Mouse	Tanaka et al. (2003)
NPB (i.c.v.)	Prolactin ↑	Rat (male)	Samson et al. (2004)
	Growth hormone ↑	
NPW23/NPB (i.t)	Inflammatory pain ↓	Rat	Yamamoto et al. (2005)
NPW/NPB (i.c.v.)	Corticosterone in circulation ↑	Rat (male)	Samson et al. (2004)
			Taylor et al. (2005)
NPW/NPB (i.p)	(Plasma level) parathyroid hormone ↑	Rat	Hochol et al. (2007)
	(Plasma level) corticosterone ↑	
	(Plasma level) testosterone ↑	
NPW/NPB (i.c.v.)	Circadian rhythm →	Rat/mouse	Our unpublished observations

**Table 2:** **Summary of behavioral phenotypes of NPBWR1 knockout mice (modified from Nagata-Kuroiwa et al., 2011)**.

Behavioral test	Parameter	Results
Open field test	Anxiety	Normal time spent in center of arena
Elevated plus maze test	Anxiety	Normal time spent and number of entries in open arms
Light-dark exploration test	Anxiety	Decrease in escape latency and time spent in light box
Porsolt forced swim test	Depression, learning helplessness	Normal time spent swimming
Prepulse inhibition test	Sensory motor reactivity	Normal percentage of prepulse inhibition
Marble burying behavior test	Compulsive behavior	Normal number of marbles buried
Cued and contextual fear conditioning test	Fear and memory	Decrease in time of freezing behavior during contextual testing while normal during auditory-cued testing
Morris water maze test	Spatial memory	Normal escape latency
Resident-intruder test	Social interaction	Abnormal social interaction (see text)
Stress-induced hyperthermia	Stress response	Exaggerated hyperthermia
Daily locomotor activity	Circadian rhythm	Normal in both light/dark cycle and constant dark condition. Normal entrainment by food or light
Sleep-wake behavior (EEG/EMG)	Sleep/wake cycle	Normal in each episode duration, times spent in each state in hourly sleep/wake analysis

### Feeding and energy homeostasis

The NPB/W system was initially thought to be involved in regulation of feeding behavior (Shimomura et al., 2002; Tanaka et al., 2003), and therefore many of the currently available reports have focused on the roles of NPB/W in this function. The first physiological study on the action of NPW showed acute hyperphagia in male rats when NPW was administered intracerebroventricularly (i.c.v.) (Shimomura et al., 2002; Tanaka et al., 2003). However, Tanaka et al. (2003) showed that the effect of NPB on feeding behavior in mice is very complex. When NPB was i.c.v. injected during the light period, no significant effect of NPB on feeding was observed (Tanaka et al., 2003). In contrast, in the dark period, i.c.v. administration of 3 nmol NPB increased feeding only within the first 2 h. A higher dose of NPB suppressed food intake in this period. After 2 h, both doses of NPB decreased food intake. Because rodents only have NPBWR1 as a receptor for NPB, the biphasic, i.e., earlier orexigenic and later anorexic, effect cannot stem from the different potency rank orders of the two peptides on NPBWR1 and NPBWR2. In fact, we also observed a similar biphasic action of NPW on feeding behavior when administered i.c.v. in mice or rats (our unpublished observation). Mondal et al. (2003) also reported anorexic effects of NPW. These findings suggest a complex role of NPB and NPW in the regulation of food intake.

Interestingly, the anorexic effect of NPB was markedly enhanced when corticotrophin-releasing factor (CRF), a known anorexic peptide, was co-administered (Tanaka et al., 2003). The i.c.v. administration of these two peptides almost completely suppressed food intake over 4 h. The biphasic effects of NPB/W on feeding behavior and the synergistic anorexic effects of NPB and CRF suggest complex roles of these peptides in the regulation of feeding behavior in relation to the stress response. The synergic effect of NPB with CRF in suppression of feeding suggests that this neuropeptide is likely to be implicated in inhibition of feeding under stressful conditions.

Continuous i.c.v. infusion of NPW was reported to suppress feeding and body weight gain over the infusion period (Mondal et al., 2003). Conversely, i.c.v. administration of anti-NPW IgG stimulated feeding, suggesting that endogenous NPW plays an inhibitory role in feeding behavior (Mondal et al., 2003). However, unlike the results of continuous i.c.v. infusion of NPW, bolus intra-PVN microinjection of NPW23 at doses ranging from 0.1 to 3 nmol increased feeding for up to 4 h, and a bolus dose ranging from 0.3 to 3 nmol was reported to increase feeding for up to 24 h (Levine et al., 2005). These observations suggest that the orexigenic versus anorectic effects of NPB/W could result from different sites of action. When these peptides are injected into the lateral ventricle, they might initially act on the PVN, followed by diffusion to other regions implicated in the suppression of feeding. Alternatively, delayed inhibition of feeding by NPB/W might result from the production of other anorectic factors that are stimulated by NPB or NPW.

The i.c.v. administration of NPW also increased body temperature and heat production (Mondal et al., 2003). These effects suggest that endogenous NPB/W might increase energy expenditure, which is consistent with the late-onset obesity seen in male *NPBWR1*^−/−^ mice and *NPB*^−/−^ mice (Ishii et al., 2003; Kelly et al., 2005).

Male *NPBWR1*^−/−^ mice have been shown to develop adult-onset obesity that progressively worsens with age and is greatly exacerbated when animals are fed a high-fat diet (Ishii et al., 2003). The obesity is caused by hyperphagia along with decreased energy expenditure. *Npbwr1*^−/−^ male mice showed decreased hypothalamic *neuropeptide Y* mRNA level and increased *proopiomelanocortin* mRNA level, a set of effects opposite to those evident in *ob/ob* mice. Furthermore, *ob/ob*;*NPBWR1*^−/−^ and *Ay/a*;*NPBWR1*^−/−^ double mutant male mice had an increased body weight compared with normal *ob/ob* or *Ay/a* male mice, suggesting that the obesity of *NPBWR1*^−/−^ mice is independent of leptin and melanocortin signaling. Female mice did not show any significant weight increase or associated metabolic defects. These data suggest a potential role for NPBWR1 in regulating energy homeostasis independent of leptin and melanocortin signaling, in a sexually dimorphic manner (Ishii et al., 2003). Consistent with the phenotype of *Npbwr1*^−/−^ mice, *Npb*^−/−^ mice were also reported to exhibit mild adult-onset obesity (Kelly et al., 2005).

### Pain regulation

Intracerebroventricular administration of NPB produces analgesia to pain induced by subcutaneous formalin injection in rats (Tanaka et al., 2003). Intrathecal (i.t.) injection of either NPW23 or NPB also decreased agitation behaviors induced by formalin injection into the paw and attenuated the level of mechanical allodynia (Yamamoto et al., 2005). The effects were not antagonized by naloxone, suggesting that this effect is not mediated through the opioid receptor system. While i.t. injection of either NPW23 or NPB did not show any effect in the hot plate test or mechanical nociceptive test, i.t. injection of either NPW23 or NPB significantly suppressed the expression of Fos-like immunoreactivity of the L4–5 spinal dorsal horn induced by formalin injection into the paw. These data suggest that spinally applied NPW/B suppressed the input of nociceptive information to the spinal dorsal horn, producing an analgesic effect on inflammatory pain, but not mechanical or thermal pain (Yamamoto et al., 2005).

Consistent with the pharmacological studies suggesting an analgesic action of NPB on inflammatory pain, *NPB*^−/−^ mice exhibited hyperalgesia to inflammatory pain, while they showed normal responses to mechanical or thermal pain (Kelly et al., 2005). These observations suggest that NPB in the brain and/or spinal cord modulates pain in a modality-specific manner. These observations suggest that NPB might play a role in the pathophysiology of allodynia. Unfortunately, however there are no available data about expression of NPW, NPW, or NPBWR1 in the spinal cord or dorsal root ganglia.

A low level of NPBWR1 receptor expression was observed in Schwann cells in both normal human and rat nerves as well as in primary rat Schwann cell cultures. Peripheral nerve samples taken from patients exhibiting inflammatory/immune-mediated neuropathy showed a marked increase of NPBWR1 receptor expression restricted to myelin-forming Schwann cells. Complementary animal models of immune-inflammatory and ligation-induced nerve injury and neuropathic pain similarly exhibited increased myelin-associated expression of NPBWR1 (Zaratin et al., 2005). These observations suggest that NPBWR1 is involved in regulation of inflammatory pain at least in part by modulating Schwann cell function.

### Neuroendocrine regulation

Central administration of NPB or NPW elevated the plasma corticosterone level in rats (Samson et al., 2004; Taylor et al., 2005). NPB also increased prolactin and decreased growth hormone levels (Samson et al., 2004). Pretreatment with a polyclonal anti-CRF antiserum or CRF antagonist completely blocked the effect of NPB or NPW to stimulate ACTH release, and significantly inhibited the effect of NPB/W on plasma corticosterone level (Samson et al., 2004; Taylor et al., 2005). These findings suggest that NPW and NPB play a role in the neuroendocrine response to stress via activation of the hypothalamus-pituitary-adrenal (HPA) axis. Consistent with these observations, whole cell patch-clamp recording from hypothalamic slice preparations showed that bath application of NPW depolarized and directly increased the spike frequency of neuroendocrine PVN neurons (Taylor et al., 2005).

A whole cell patch-clamp study showed that NPW23 exhibited effects on oxytocin, vasopressin, and thyrotrophin-releasing hormone neurons in the PVN, although both depolarizing and hyperpolarizing effects were observed in each of these cell groups (Price et al., 2009). Corticotrophin-releasing hormone cells were unaffected. Further subdivision of chemically phenotyped cell groups into magnocellular, neuroendocrine, or pre-autonomic neurons, using their electrophysiological fingerprints, revealed that neurons projecting to medullary and spinal targets were predominantly inhibited by NPW, whereas those that projected to the median eminence or neural lobe showed almost equivalent numbers of depolarizing and hyperpolarizing cells (Price et al., 2009).

The intracellular mechanisms of these electrophysiological effects of NPW have remained unclear. Since NPBWR1 primarily couples to Gi subclass of G-proteins, activation of this receptor should lead to inhibition of neurons. Depolarizing effect of NPW described in above mentioned report might be indirect effect due to inhibition of inhibitory interneurons of recorded cells.

### Autonomic regulation

Intracerebroventricular administration of NPW to rats was reported to increase arterial blood pressure (ABP), heart rate (HR), and plasma catecholamine concentration (Yu et al., 2007). The same report showed that most of the PVN neurons were excited, while a subset of a smaller population of PVN neurons was inhibited by NPW30, although the chemical identities of these neurons were not determined. These observations suggest that NPB/W modulate PVN neuronal activities, which play an important role in regulation of the autonomic nervous system as well as the HPA axis. The expression of NPB mRNA in the PVN suggests that NPB is more likely to be involved in HPA axis regulation. However, *NPBWR1*^−/−^ mice have normal blood pressure and HR in a basal state (Nagata-Kuroiwa et al., 2011). The physiological relevance of the NPB/W system in regulation of the autonomic nervous system remains unclear.

### Sleep and wakefulness

Neuropeptide B is reported to induce slow wave sleep in mice when injected intracerebroventricularly (Hirashima et al., 2011). However, since *NPBWR1^−^/−* mice did not show any sleep abnormality, the biological relevance of this finding remains unclear.

### Emotion and behavior

*Npbwr1* mRNA is abundantly expressed in discrete brain regions in rodents, including the hypothalamus (dorsomedial hypothalamus and suprachiasmatic nucleus), hippocampus, VTA, and extended amygdala (the CeA and bed nucleus of the stria terminalis; BNST) (Lee et al., 1999; Tanaka et al., 2003). These regions are implicated in mood and emotion. Especially, the particularly strong expression of *Npbwr1* in the CeA and BNST, together with the robust projections of NPW-containing axons to these regions (Kitamura et al., 2006), suggest that this receptor might be an important modulator or regulator of the output signal from the extended amygdala, which is a well-defined subcortical nuclear group that is the center of emotion including fear (Phelps and LeDoux, 2005).

A sensory stimulus associated with an aversive outcome will change neural transmission in the amygdala to produce somatic, autonomic, and endocrine signs of fear, as well as increased attention to that stimulus. Fear learning involves the lateral and basolateral amygdala (BLA), where the association between incoming sensory stimuli leads to potentiation of synaptic transmission. The BLA receives sensory information from the thalamus, hippocampus, and cortex, and then activates or modulates synaptic transmission in target areas appropriate for the reinforcement signal with which the sensory information has been associated. The BLA projects to the CeA and BSNT, whose efferents to the hypothalamus and brainstem trigger the expression of fear. Histological and electrophysiological studies have shown that NPBWR1 acts as an inhibitory regulator on a subpopulation of GABAergic neurons in the lateral division of the CeA and terminates stress responses (Nagata-Kuroiwa et al., 2011). These anatomical and functional findings suggest that the NPB/W systems might have important roles in the modulation of output from the extended amygdala.

The role of NPBWR1 in social behavior was recently investigated using *Npbwr1*^−/−^ mice (Nagata-Kuroiwa et al., 2011). When presented with an intruder mouse, *Npbwr1*^−/−^ mice showed impulsive contact with the strange mice, produced more intense approaches toward them, and had longer contact and chasing time along with greater and sustained elevation of HR and blood pressure compared to wild type mice. *Npbwr1*^−/−^ mice also showed increased autonomic and neuroendocrine responses to various physical stresses, suggesting that NPBWR1 might play a role in coping with stress.

Another interesting phenotype of *NPBWR1*^−/−^ mice is the impairment of contextual fear conditioning and inversion of “safety conditioning.” In the safety conditioning paradigm, mice received unpaired presentations of a CS (tone) and a US (electrical shock). Because the shock never occurs during the CS, mice typically learn to treat the CS as a safety signal, so that fear-related behavior is inhibited during presentation of the CS. *Npbwr1*^−/−^ mice show an inversion of this learning in that the safety-trained CS elicits fear rather than a reduction in fear. This observation suggests that *Npbwr1*^−/−^ mice undergo trace conditioning rather than safety conditioning in this procedure, in that they associate the CS and US across a long trace interval, while the control mice treat CS and US as unpaired. These results suggest that *Npbwr1*^−/−^ mice are more stimulus-bound, meaning that they preferentially attend to discrete stimuli, to the exclusion of more complex, conjunctive stimuli such as context. This hypothesis would also explain the deficit in contextual fear conditioning. To determine the neural mechanisms and the possible developmental and/or extra-amygdalar origins of the phenotype, further investigation using spatially restricted knockout mice and/or genetic rescue of the phenotype of these mice by expressing NPBWR1 in a region-specific manner is needed.

The human *NPBWR1* gene has a frequent single nucleotide polymorphism at nucleotide 404 (SNP rs33977775) in the coding region (404A >T). Importantly, this polymorphism causes an amino acid substitution (Y135F) within the highly conserved DRY motif of G-protein-coupled receptors at the junction of the third transmembrane domain and second intracellular loop, which is known to play an important role in G-protein coupling. Recently, Watanabe et al. (2012) demonstrated that this change partially impairs receptor function as measured by inhibition of cAMP production. Furthermore, this variation affects the emotional response to stimuli showing human faces with four categories of emotional expression (anger, fear, happiness, and neutral). The subject’s emotional levels at seeing these faces were rated on scales of hedonic valence, emotional arousal, and dominance (V–A–D). A significant genotype difference was observed in valence evaluation; the 404AT group (position 404 in one allele is replaced by T) perceived the face as more pleasant than did the 404AA group (position 404 in both alleles of *NPBWR1* is A), regardless of the category of facial expression. The 404AT group tended to feel less submissive to an angry face than did the 404AA group. These results suggest that a single nucleotide polymorphism of NPBWR1 seems to affect human behavior in a social context. Future studies using functional brain imaging of human subjects are needed to clarify the mechanisms of the effects of the polymorphisms.

### Effects on circadian rhythm

The abundant expression of NPBWR1 in the suprachiasmatic nucleus suggests the possibility that this neuropeptides/receptor system has a role in regulating circadian rhythm (Lee et al., 1999; Tanaka et al., 2003; Singh et al., 2004). However, we did not observe any effects of NPB/W on circadian activity in rats or mice when administered by i.c.v. injection. *Npbwr1*^−/−^ mice displayed a normal circadian pattern of behavior in both light-dark and constant dark conditions (Uchio et al., 2009). Both light-entrainable and food-entrainable oscillation were also normal in these mice (our unpublished observations). The function of NPBWR1 in the SCN remains unclear thus far.

### Peripheral actions

Expression of *Npb*, *Npw*, and *Npbwr1* mRNA in both the adrenal cortex and adrenal medulla has been reported (Andreis et al., 2005; Mazzocchi et al., 2005; Hochol et al., 2007). NPB and NPW were shown to pharmacologically stimulate adrenal glucocorticoid secretion by an ACTH-independent mechanism. It was also reported that NPW stimulates *in vitro* aldosterone secretion by enhancing the release of medullary catecholamines, which activate beta-adrenoceptors located on zona glomerulosa cells (Hochol et al., 2007).

Bolus intraperitoneal (i.p.) injection of NPB or NPW increased the plasma levels of parathyroid hormone, corticosterone, and testosterone. NPB was also reported to increase the blood concentration of thyroxine, and NPW was shown to increase ACTH and estradiol levels. These findings suggest that NPB and NPW play a role in regulation of the endocrine system (Hochol et al., 2006).

The existence of NPW in rat gastric antral cells was reported. The level of NPW in the stomach was decreased in fasted animals, while it was increased by re-feeding (Mondal et al., 2003), which is consistent with the notion that NPW may act as a suppressant of feeding. However, we did not observe any effects on feeding in mice when NPB or NPW was intravenously administered, suggesting that peripheral NPB/W has limited, if any, significant role in modulating feeding behavior (our unpublished observations).

Neuropeptide B and NPW are reported to inhibit proliferative activity of rat calvarial osteoblast-like (ROB) cells (Ziolkowska et al., 2009). However, the physiological relevance of this finding is unknown, and requires further investigation.

## Conclusion

Neuropeptide B and NPW are likely to be multi-tasking factors. Both *Npb*^−/−^ and *Npbwr1*^−/−^ mice show late-onset obesity and hyperphagia, suggesting that the endogenous NPB-NPBWR1 pathway negatively regulates feeding behavior and positively regulates energy expenditure (Ishii et al., 2003; Kelly et al., 2005). This notion is further supported by several pharmacological studies that showed that i.c.v. NPB/W increased heat production and sympathetic outflow (Yu et al., 2007). However, the biphasic (early orexigenic followed by anorexic) effects of NPB/NPW suggest complex actions of NPB and NPW in the regulation of food intake.

Many studies have also shown that the NPB/W system is involved in the modulation of inflammatory pain. Consistent with these results, *Npb*^−/−^ mice are hypersensitive to inflammatory pain but display no significant difference in chemical or thermal pain. These data together strongly support a physiological role of central NPB in pain regulation, and agonists for NPBWR1 or NPBWR2 might be good candidates for analgesic drugs for chronic inflammatory pain.

Finally, the strong, discrete expression of NPBWR1 in the extended amygdala and abundant projections of NPW fibers in these regions suggest that this neuropeptide system has a role in the regulation of fear and anxiety. The CeA and BNST have been implicated in a variety of emotional functions including expression of fear, modulation of memory, and mediation of social communication (Davis and Shi, 1999) (Figure 3). Therefore, the expression of NPBWR1 in the CeA and BNST suggests modulatory roles of this receptor in these functions. Recent studies in *Npbwr1*^−/−^ mice support this concept (Table 2). Furthermore, a recent report suggested that a human polymorphism in the *NPBWR1* gene might affect emotional responses to facial expressions (Watanabe et al., 2012). In daily life, people show various emotional reactions to others depending on their personalities, even in the same situation. NPBWR1 may provide part of the cause for such differences in reactions in social interactions.

## Conflict of Interest Statement

The author declares that the research was conducted in the absence of any commercial or financial relationships that could be construed as a potential conflict of interest.

## References

[B1] Andreis P. Rucinski M. Neri G. Conconi M. Petrelli L. Parnigotto P. (2005). Neuropeptides B and W enhance the growth of human adrenocortical carcinoma-derived NCI-H295 cells by exerting MAPK p42/p44-mediated proliferogenic and antiapoptotic effects. Int. J. Mol. Med. 16, 1021–1028 16273281

[B2] Anthony Romero F. Hastings N. B. Moningka R. Guo Z. Wang M. Di Salvo J. (2012). The discovery of potent antagonists of NPBWR1 (GPR7). Bioorg. Med. Chem. Lett. 22, 1014–1018 10.1016/j.bmcl.2011.11.126 22197390

[B3] Brezillon S. Lannoy V. Franssen J. D. Le Poul E. Dupriez V. Lucchetti J. (2003). Identification of natural ligands for the orphan G protein-coupled receptors GPR7 and GPR8. J. Biol. Chem. 278, 776–783 10.1074/jbc.M206396200 12401809

[B4] Davenport A. Singh G. (2005). Neuropeptide W/Neuropeptide B Receptors – NPBW1. IUPHAR Receptor database. [10.1786/080844542445].

[B5] Davis M. Shi C. (1999). Extended amygdala and basal forebrain. Ann. N. Y. Acad. Sci. 877, 281–291 10.1111/j.1749-6632.1999.tb09273.x 10415655

[B6] Dun S. L. Brailoiu G. C. Yang J. Chang J. K. Dun N. J. (2003). Neuropeptide W-immunoreactivity in the hypothalamus and pituitary of the rat. Neurosci. Lett. 349, 71–74 10.1016/S0304-3940(03)00804-8 12946555

[B7] Fujii R. Yoshida H. Fukusumi S. Habata Y. Hosoya M. Kawamata Y. (2002). Identification of a neuropeptide modified with bromine as an endogenous ligand for GPR7. J. Biol. Chem. 277, 34010–34016 10.1074/jbc.M205883200 12118011

[B8] Hirashima N. Tsunematsu T. Ichiki K. Tanaka H. Kilduff T. S. Yamanaka A. (2011). Neuropeptide B induces slow wave sleep in mice. Sleep 34, 31–37 21203369 10.1093/sleep/34.1.31 PMC3001792

[B9] Hochol A. Belloni A. Rucinski M. Ziolkowska A. Di Liddo R. Nussdorfer G. (2006). Expression of neuropeptides B and W and their receptors in endocrine glands of the rat. Int. J. Mol. Med. 18, 1101–1106 17089014

[B10] Hochol A. Tortorella C. Ricinski M. Ziolkowska A. Nussdorfer G. Malendowicz L. (2007). Effects of neuropeptides B and W on the rat pituitary-adrenocortical axis: in vivo and in vitro studies. Int. J. Mol. Med. 19, 207–211 17203193

[B11] Hondo M. Ishii M. Sakurai T. (2008). The NPB/NPW neuropeptide system and its role in regulating energy homeostasis, pain, and emotion. Results Probl. Cell Differ. 46, 239–256 10.1007/400_2007_056 18204824

[B12] Ishii M. Fei H. Friedman J. M. (2003). Targeted disruption of GPR7, the endogenous receptor for neuropeptides B and W, leads to metabolic defects and adult-onset obesity. Proc. Natl. Acad. Sci. U.S.A. 100, 10540–10545 10.1073/pnas.1334189100 12925742 PMC193597

[B13] Jackson V. R. Lin S. H. Wang Z. Nothacker H. P. Civelli O. (2006). A study of the rat neuropeptide B/neuropeptide W system using in situ techniques. J. Comp. Neurol. 497, 367–383 10.1002/cne.20989 16736466

[B14] Kelly M. A. Beuckmann C. T. Williams S. C. Sinton C. M. Motoike T. Richardson J. A. (2005). Neuropeptide B-deficient mice demonstrate hyperalgesia in response to inflammatory pain. Proc. Natl. Acad. Sci. U.S.A. 102, 9942–9947 10.1073/pnas.0503795102 15983370 PMC1174999

[B15] Kitamura Y. Tanaka H. Motoike T. Ishii M. Williams S. C. Yanagisawa M. (2006). Distribution of neuropeptide W immunoreactivity and mRNA in adult rat brain. Brain Res. 1093, 123–134 10.1016/j.brainres.2006.03.041 16697979

[B16] Lee D. K. Nguyen T. Porter C. A. Cheng R. George S. R. O’Dowd B. F. (1999). Two related G protein-coupled receptors: the distribution of GPR7 in rat brain and the absence of GPR8 in rodents. Brain Res. Mol. Brain Res. 71, 96–103 10.1016/S0169-328X(99)00171-0 10407191

[B17] Levine A. Winsky-Sommerer R. Huitron-Resendiz S. Grace M. de Lecea L. (2005). Injection of neuropeptide W into paraventricular nucleus of hypothalamus increases food intake. Am. J. Physiol. Regul. Integr. Comp. Physiol. 288, R1727–R1732 10.1152/ajpregu.00638.2003 15886360

[B18] Lucyk S. Miskolzie M. K. G. (2005). NMR conformational analyses on (des-bromo) neuropeptide B [1-23] and neuropeptide W [1-23]: the importance of alpha-helices, a cation-pi interaction and a beta-turn. J. Biomol. Struct. Dyn. 23, 77–90 10.1080/07391102.2005.10507049 15918679

[B19] Mazzocchi G. Rebuffat P. Ziolkowska A. Rossi G. Malendowicz L. Nussdorfer G. (2005). G protein receptors 7 and 8 are expressed in human adrenocortical cells, and their endogenous ligands neuropeptides B and w enhance cortisol secretion by activating adenylate cyclase- and phospholipase C-dependent signaling cascades. J. Clin. Endocrinol. Metab. 90, 3466–3471 10.1210/jc.2004-2132 15797961

[B20] Mondal M. S. Yamaguchi H. Date Y. Shimbara T. Toshinai K. Shimomura Y. (2003). A role for neuropeptide W in the regulation of feeding behavior. Endocrinology 144, 4729–4733 10.1210/en.2003-0536 12959997

[B21] Nagata-Kuroiwa R. Furutani N. Hara J. Hondo M. Ishii M. Abe T. (2011). Critical role of neuropeptides B/W receptor 1 signaling in social behavior and fear memory. PLoS ONE 6:e16972 10.1371/journal.pone.0016972 21390312 PMC3044739

[B22] O’Dowd B. F. Scheideler M. A. Nguyen T. Cheng R. Rasmussen J. S. Marchese A. (1995). The cloning and chromosomal mapping of two novel human opioid-somatostatin-like receptor genes, GPR7 and GPR8, expressed in discrete areas of the brain. Genomics 28, 84–91 10.1006/geno.1995.1109 7590751

[B23] Phelps E. A. LeDoux J. E. (2005). Contributions of the amygdala to emotion processing: from animal models to human behavior. Neuron 48, 175–187 10.1016/j.neuron.2005.09.025 16242399

[B24] Price C. J. Samson W. K. Ferguson A. V. (2009). Neuropeptide W has cell phenotype-specific effects on the excitability of different subpopulations of paraventricular nucleus neurones. J. Neuroendocrinol. 21, 850–857 10.1111/j.1365-2826.2009.01904.x 19686447 PMC3861898

[B25] Samson W. K. Baker J. R. Samson C. K. Samson H. W. Taylor M. M. (2004). Central neuropeptide B administration activates stress hormone secretion and stimulates feeding in male rats. J. Neuroendocrinol. 16, 842–849 10.1111/j.1365-2826.2004.01239.x 15500544

[B26] Schulz S. Stumm R. Hollt V. (2007). Immunofluorescent identification of neuropeptide B-containing nerve fibers and terminals in the rat hypothalamus. Neurosci. Lett. 411, 67–71 10.1016/j.neulet.2006.10.015 17067739

[B27] Shimomura Y. Harada M. Goto M. Sugo T. Matsumoto Y. Abe M. (2002). Identification of neuropeptide W as the endogenous ligand for orphan G-protein-coupled receptors GPR7 and GPR8. J. Biol. Chem. 277, 35826–35832 10.1074/jbc.M205337200 12130646

[B28] Singh G. Maguire J. J. Kuc R. E. Fidock M. Davenport A. P. (2004). Identification and cellular localisation of NPW1 (GPR7) receptors for the novel neuropeptide W-23 by [125I]-NPW radioligand binding and immunocytochemistry. Brain Res. 1017, 222–226 10.1016/j.brainres.2004.03.079 15261118

[B29] Tanaka H. Yoshida T. Miyamoto N. Motoike T. Kurosu H. Shibata K. (2003). Characterization of a family of endogenous neuropeptide ligands for the G protein-coupled receptors GPR7 and GPR8. Proc. Natl. Acad. Sci. U.S.A. 100, 6251–6256 10.1073/pnas.2233339100 12719537 PMC156358

[B30] Taylor M. Yuill E. Baker J. Ferri C. Ferguson A. Samson W. (2005). Actions of neuropeptide W in paraventricular hypothalamus: implications for the control of stress hormone secretion. Am. J. Physiol. Regul. Integr. Comp. Physiol. 288, R270–R275 10.1152/ajpregu.00781.2004 15345475

[B31] Tim van B. Brian E. H. Deirdre K. L. Kathleen M. K. Kazushige T. Emilio P. (1995). Receptor-tyrosine-kinase-and Gβγ-mediated MAP kinase activation by a common signalling pathway. Nature 376, 781–784 10.1038/376781a0 7651538

[B32] Uchio N. Doi M. Matsuo M. Yamazaki F. Mizoro Y. Hondo M. (2009). Circadian characteristics of mice depleted with GPR7. Biomed. Res. 30, 357–364 10.2220/biomedres.30.357 20051645

[B33] Watanabe N. Wada M. Irukayama-Tomobe Y. Ogata Y. Tsujino N. Suzuki M. (2012). A single nucleotide polymorphism of the neuropeptide B/W receptor-1 gene influences the evaluation of facial expressions. PLoS ONE 7:e35390 10.1371/journal.pone.0035390 22545105 PMC3335863

[B34] Yamamoto T. Saito O. Shono K. Tanabe S. (2005). Anti-hyperalgesic effects of intrathecally administered neuropeptide W-23, and neuropeptide B, in tests of inflammatory pain in rats. Brain Res. 1045, 97–106 10.1016/j.brainres.2005.03.027 15910767

[B35] Yu N. Kunitake T. Kato K. Nakazato M. Kannan H. (2007). Effects of intracerebroventricular administration of neuropeptide W30 on neurons in the hypothalamic paraventricular nucleus in the conscious rat. Neurosci. Lett. 15, 40–145 10.1016/j.neulet.2007.01.048 17300871

[B36] Zaratin P. Quattrini A. Previtali S. Comi G. Hervieu G. Scheideler M. (2005). Schwann cell overexpression of the GPR7 receptor in inflammatory and painful neuropathies. Mol. Cell. Neurosci. 28, 55–63 10.1016/j.mcn.2004.08.010 15607941

[B37] Ziolkowska A. Rucinski M. Tyczewska M. Malendowicz L. K. (2009). Neuropeptide B (NPB) and neuropeptide W (NPW) system in cultured rat calvarial osteoblast-like (ROB) cells: NPW and NPB inhibit proliferative activity of ROB cells. Int. J. Mol. Med. 24, 781–787 19885618 10.3892/ijmm_00000292

